# Complete genome and gene expression analyses of *Asaia bogorensis* reveal unique responses to culture with mammalian cells as a potential opportunistic human pathogen

**DOI:** 10.1093/dnares/dsv018

**Published:** 2015-09-10

**Authors:** Mikihiko Kawai, Norie Higashiura, Kimie Hayasaki, Naruhei Okamoto, Akiko Takami, Hideki Hirakawa, Kazunobu Matsushita, Yoshinao Azuma

**Affiliations:** 1 Faculty of Biology-Oriented Science and Technology, Kindai University, Kinokawa, Wakayama, Japan; 2 Advanced Low Carbon Technology Research and Development Program (ALCA), Japan Science and Technology Agency (JST), Tokyo, Japan; 3 Kazusa DNA Institute, Kisarazu, Chiba, Japan; 4 Department of Biological Chemistry, Faculty of Agriculture, Yamaguchi University, Yamaguchi, Yamaguchi, Japan

**Keywords:** genomics, transcriptome, ubiquinol terminal oxidase, opportunistic pathogen, acetic acid bacteria

## Abstract

*Asaia bogorensis*, a member of acetic acid bacteria (AAB), is an aerobic bacterium isolated from flowers and fruits, as well as an opportunistic pathogen that causes human peritonitis and bacteraemia. Here, we determined the complete genomic sequence of the *As. bogorensis* type strain NBRC 16594, and conducted comparative analyses of gene expression under different conditions of co-culture with mammalian cells and standard AAB culture. The genome of *As. bogorensis* contained 2,758 protein-coding genes within a circular chromosome of 3,198,265 bp. There were two complete operons encoding cytochrome *bo_3_*-type ubiquinol terminal oxidases: *cyoABCD-1* and *cyoABCD-2*. The *cyoABCD-1* operon was phylogenetically common to AAB genomes, whereas the *cyoABCD-2* operon belonged to a lineage distinctive from the *cyoABCD-1* operon. Interestingly, *cyoABCD-1* was less expressed under co-culture conditions than under the AAB culture conditions, whereas the converse was true for *cyoABCD-2. Asaia bogorensis* shared pathogenesis-related genes with another pathogenic AAB, *Granulibacter bethesdensis*, including a gene coding pathogen-specific large bacterial adhesin and additional genes for the inhibition of oxidation and antibiotic resistance. Expression alteration of the respiratory chain and unique hypothetical genes may be key traits that enable the bacterium to survive under the co-culture conditions.

## 1. Introduction

Acetic acid bacteria (AAB) are obligate aerobes, and presently comprise 14 genera assigned to the family Acetobacteraceae, including *Acetobacter* (*Ac.*), *Gluconobacter* (*Go.*), *Gluconacetobacter* (*Ga.*), *Granulibacter* (*Gr.*), *Komagataeibacter* (*Ko.*, former *Gluconacetobacter*), *Asaia* (*As.*), *Acidomonas* (*Am.*), *Kozakia*, *Swaminathania*, *Saccharibacter*, *Neoasaia*, *Tanticharoenia*, *Ameyamaea*, and *Neokomagataea.*^1,2^ AAB incompletely oxidize a variety of sugars and alcohols, and accumulate large amounts of the corresponding oxidation products in their environment or culture media. These traits of AAB, known as oxidative fermentation, are utilized in the industrial production of vinegar, l-sorbose, and bacterial cellulose.

Oxidative fermentation reactions occur through a variety of primary dehydrogenases in the periplasmic side of the inner cytoplasmic membrane. Electrons that are generated are transferred to ubiquinone and the reduced ubiquinol is utilized by terminal oxidases in the electron transfer chains to reduce oxygen to water and harness the energy to translocate protons across the cytoplasmic membrane.^3^ There are two types of terminal oxidases in AAB: cytochrome *ba_3_*/*bo_3_*-type ubiquinol oxidase and *bd*-type oxidase, which consist of four subunits encoded by the *cyoABCD* operon (synonym: *cyaBACD*) and two subunits encoded by *cydAB* genes, respectively. Cytochrome *ba_3_*/*bo_3_*-type ubiquinol oxidase is a member of the haeme–copper oxidase superfamily, which includes cytochrome *c* oxidase in aerobic respiration of mitochondria and aerobes.^4^

*Asaia bogorensis* was first isolated from tropical flowers, and is an unusual member of AAB that oxidizes acetate and lactate to carbon dioxide and water, but not ethanol to acetic acid.^5^ A strain of *As. bogorensis* isolated from a flower of the orchid tree *Bunga bauhinia* in Bogor, Indonesia, was assigned to a type strain (=NBRC 16594^T^= NRIC 0311^T^= JCM 10569^T^).^5^ Based on DNA sequences of 16S rRNA genes, *Asaia* is phylogenetically located near *Gluconobacter* and *Acetobacter*, but is distant from the genera *Granulibacter* and *Gluconacetobacter*.^5,6^ Biochemical analysis revealed that *As. bogorensis* contains sugar- and sugar alcohol-oxidizing enzymes that are specific to the respiratory chain, such as quinoprotein glycerol dehydrogenase; however, it lacks quinoprotein alcohol dehydrogenase (ADH).^7^ In addition, *As. bogorensis* was shown to contain a cytochrome *bo_3_*-type ubiquinol oxidase as the sole terminal oxidase in the respiratory chain.^7^

*Asaia bogorensis* can also be an opportunistic pathogen in humans. *Asaia bogorensis* was first reported as an opportunistic human pathogen when it was isolated from the peritoneal fluids of a patient on peritoneal dialysis who showed a consistent clinical status of infectious peritonitis with end-stage renal disease.^8^ In addition, it was reported that *As. bogorensis* caused bacteraemia in patients with a history of intravenous-drug abuse.^9^ Clinical isolates were highly resistant to antibiotics that were routinely tested against gram-negative bacteria, but not against gentamicin or doxycycline.^9,10^ As reported about infectious diseases of AAB in humans,^6,11,12^ it is important to comprehend the genetic bases for the pathogenicity of AAB.

Gene contents of organisms may explain their phenotypes, as well as abilities to respond to variety of environmental stresses, and provide us with information regarding their potential to adapt to new ecological niches. Complete genomic DNA sequences from five AAB species have been published for their industrial, agricultural, and clinical significances, including *Acetobacter pasteurianus*,^13,14^ *Gluconobacter oxydans*,^15,16^ *Komagataeibacter medellinensis* (former *Gluconacetobacter xylinus*),^17^ *Gluconacetobacter diazotrophicus*,^18,19^ and *Granulibacter bethesdensis*.^20^ To establish a genetic platform for the features of AAB, many draft genomes of unique AAB species have been published, such as *Acetobacter pomorum* and *Asaia platycodi* isolated from insect guts,^21,22^ as well as the methylotroph AAB, *Acidomonas methanolica*.^23^

To elucidate genes associated with the unique biochemical capabilities of *As. bogorensis* and release its genetic information for further analyses of its pathogenic strains, we here determined the complete genome sequence of a type strain of *As. bogorensis*, NBRC 16594, which is a natural but not clinical isolate. Its gene content was compared with those of other AAB genomes, and the *As. bogorensis* transcriptome was compared under different culture conditions.

## 2. Materials and methods

### 2.1. Genome sequencing

*Asaia bogorensis* strain NBRC 16594 (=NRIC 0311 = JCM 10569) was obtained from the NITE Biological Resource Center (NBRC, Japan). The genomic DNA sequence of *As. bogorensis* was determined by a whole-genome shotgun strategy, using a method described previously with slight modifications.^23^ Briefly, 4.2 µg of genomic DNA was extracted from *As. bogorensis* that was cultured aerobically in a YPGD medium (1.0% yeast extract, 1.0% polypeptone, 2.0% glycerol, and 0.5% glucose) at 30°C. After DNA fragmentation and isolation (average fragment length 189 bp), 6,345,662 reads (75 bases per read) were generated using the GAIIx sequencing system (Illumina, San Diego, CA, USA). Short DNA reads were assembled using the Velvet 1.0.3 software. Among different *k*-mers (minimum-overlapping length) that were tested, 49 *k*-mer in size provided the best result in terms of N50, resulting in 50 contigs, with a total of 3,173,055 bp (coverage: 116×). Seventy-two primers were prepared based on relatively longer 36 contigs out of the 50 ones, and DNA amplification by PCR was performed using all combinations of both ends of the contigs. DNA sequences of the fragments amplified were determined at least once in both strands by direct sequencing of PCR products using an ABI Prism 3100 Genetic Analyzer (Life Technologies). After all the gaps were filled with appropriate qualities, assembling of the genome DNA was validated by gel electrophoresis of its genomic DNA and re-mapping of the short DNA reads, resulted in no inconsistency in the assembling. No plasmids were detected in this process.

### 2.2. Gene finding and annotation

Protein-coding gene finding was performed using three programmes: GeneMarkS,^24^ MetaGeneAnnotator,^25^ and GLIMMER3.^26^ Protein-coding genes are defined when genomic loci were predicted as protein-coding genes (i) by two or more programmes, or (ii) by only one programme but supported by TIGRFAMs hits,^27^ or by distinctive RNA expression. The start positions of the genes were determined in two ways: (i) the same position was predicted as a start position by two or more programmes, or (ii) when all three programmes differently predicted start positions, the preferred was chosen from the software in the following order: GeneMarkS, MetaGeneAnnotator, and GLIMMER3. Prediction of rRNA and tRNA genes was performed using RNAmmer^28^ and tRNAscan-SE,^29^ respectively. Gene products were annotated on the basis of similar proteins of *Am. methanolica*^23^ and *Ko. medellinensis*,^17^ or most similar proteins in databases using the BLASTP program,^30^ as well as referring to conserved protein families that were predicted using the Hmmer3 program^31^ with a noise cut-off option (--cut_nc) against TIGRFAMs^27^ and PFAM^32^ databases.

### 2.3. Bioinformatics analyses

To analyse a taxonomic distribution of specific genes, a platform phylogenetic tree with whole prokaryotic organisms was illustrated using 42 single-copy genes conserved among *As. bogorensis* and the complete genome sequences of 705 prokaryotic species representing 705 different genera, including 80 archaea and 625 bacteria, using a previously described method (Supplementary Tables S1 and S2).^33,34^ The 42 genes were selected on the basis of the following two requirements: (i) the gene should be conserved among >95% of both archaea and bacteria and (ii) average copy numbers of the gene in both archaea and bacteria should be 1.10 or lower. The 42 gene products were aligned by MAFFT^35^ and concatenated, and phylogenetic distances of the species were then calculated using the FastTree program.^36^

Phylogenetic profiles were constructed by allocation of each gene in whole genomes of six AAB, including *As. bogorensis*, *Gr. bethesdensis*, *Ac. pasteurianus*, *Go. oxydans*, *Ko. medellinensis*, and *Ga. diazotrophicus*, to a certain gene family at the amino acid level using the Hmmer3 program^31^ on TIGRFAMs^27^ and PFAM^32^ databases. To predict functional and evolutionary interactions among gene families, the gene families were classified into categories, such as gene families existing in only *As. bogorensis* but not in the other five AAB, ones in only pathogenic AAB (*As. bogorensis* and *Gr. bethesdensis*), and ones shared with AAB isolated from natural samples by a mean in which not only the presence or absence of a protein family, but also the copy number of paralogous genes in each AAB genome belonging to a protein family was considered.^37^ Statistical significance of this categorization was assessed using a Wilcoxon signed-rank test, which takes into account the magnitude of the observed differences.

### 2.4. Total RNA preparation

*Asaia bogorensis* was grown under three different conditions. For cells prepared under ‘co-culture conditions’, HEK293 cells (Human Embryonic Kidney 293 cells) and McCoy cells (mouse fibroblast cells) were pre-cultured in 2 ml of Dulbecco's Modified Eagle Medium F-12 (DMEM, Sigma-Aldrich) with 5% foetal calf serum (FCS, Cansera) without antibiotics in six-well plates until 60% confluence was achieved. *Asaia bogorensis* was cultured in YPGD at 30°C until OD_600_ = 1.0, and was then diluted 10 times with DMEM with 5% FCS (no antibiotics) and incubated at 37°C with vigorous shaking at 200 rpm until OD_600_ = 1.0 (∼1 day). The supernatant of the mammalian cells were exchanged with 2 ml of the pre-cultured *As. bogorensis*, and the co-culture was incubated at 37°C for 8 h at 5% CO_2_ without agitation. The supernatant was centrifuged at 200 × *g* at 4°C for 5 min, and the mammalian cells were removed from the culture. Bacterial cells were then collected from the supernatant by centrifugation at 25°C for 5 min at 17,860 × *g*. For cells prepared under ‘tissue culture conditions’, 2 ml of the pre-cultured *As. bogorensis* was added to a six-well plate without mammalian cells, and the cells were treated in the same way as cells prepared under ‘co-culture conditions’. As a control condition (‘AAB condition’), *As. bogorensis* cells were cultured in YPGD at 30°C with aeration at 200 rpm until OD_600_ = 1.0 was achieved.

### 2.5. Transcriptome analysis

Total RNA was isolated from the bacterial cells using the RNeasy Protect Bacteria Mini Kit (QIAGEN, Germantown, MD, USA), and sequenced using HiSeq2000 (Illumina) according to the manufacturer's protocols, with the exception of a treatment to decrease a copious amount of cDNA derived from rRNA using a duplex-specific nuclease.^38^ After base calling and quality filtering using CASAVA (Illumina), the sequence was mapped to the *As. bogorensis* genomic DNA sequence and processed using the Bowtie^39^ and SAMtools software package.^40^ The number of reads that were mapped for each gene was calculated using the BedTools software.^41^ Reads that were mapped to 16S and 23S rRNA genes were omitted from further analyses. Genes that were differentially expressed between two sets of samples were estimated using the edgeR package^42^ after normalization using the TCC package.^43^ For cells that were treated under ‘co-culture conditions’, four samples (two samples containing HEK293 and two samples containing McCoy cells) were prepared from independent cultures. Adjustments of false discovery rates for *P*-values were performed for multiple comparisons using a multi-test correction. All statistical analyses were conducted using the R statistical package. After data averaging and normalization, Reads per kilobase gene per million reads (RPKM) values were used for graphical presentations.

## 3. Results

### 3.1. General features

**Figure 1.**
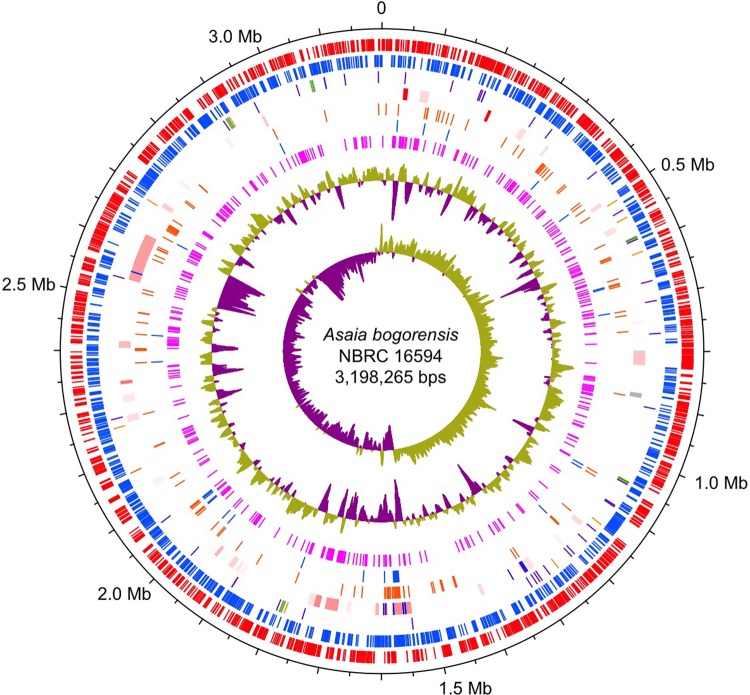
Circular representation of the *As. bogorensis* NBRC 16594 genome. From the outermost concentric circles: protein-coding sequences clockwise (red) and anti-clockwise (blue); tRNA genes (purple), rRNA genes (green), and other non-coding RNA genes (orange); transposases (blue) and the horizontally acquired region (high probability to low were shown in red to grey); genes present only in *As. bogorensis* and *Gr. bethesdensis* but absent in three acetate forming AAB (*Ac. pasteurianus*, *Ko. medellinensis*, and *Go. oxydans*) (orange); genes present in *As. bogorensis* and *Gr. bethesdensis* (blue); genes present only in *As. bogorensis* (magenta). The inner histograms show a plot of clockwise GC content and GC-skew (high and low regions in yellow green and violet, respectively). Horizontally acquired regions were predicted using Alien_hunter.^44^

**Figure 2.**
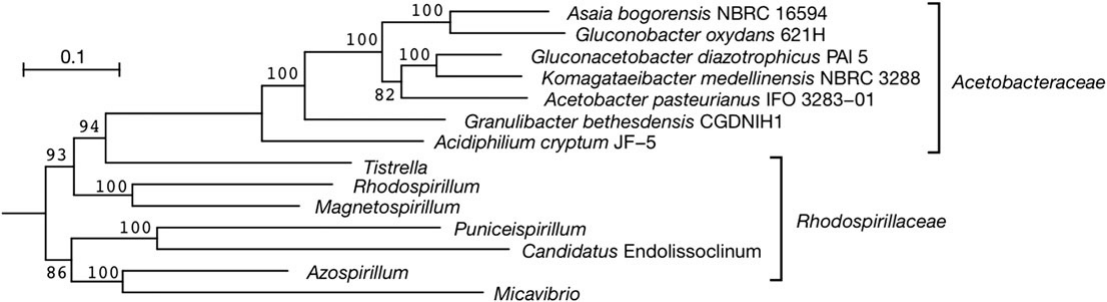
Phylogenetic tree based on 42 prokaryotic universal genes. Amino acid sequences of 42 conserved prokaryotic genes were concatenated and aligned. Numbers on branching points are confidence values of the Shimodaira–Hasegawa test (SH-test). Scale bar indicates substitutions per amino acid residue (change/amino acid site).

The complete genome sequence of *As. bogorensis* NBRC 16594 consisted of a single, circular chromosome of 3,198,265 bp with a GC content of 59.8% without plasmids (Table 1 and Fig. 1). In general, the *As. bogorensis* genome showed similar features to other complete genomes of AAB (Table 1). However, *As. bogorensis* and another AAB, which is also a human pathogen, *Gr. bethesdensis*, shared a few interesting traits, including a lack of plasmids and few transposase genes in the genomes (Table 1). Phylogenetic analysis based on 42 concatenated prokaryotic universal genes illustrated a phylogenetic tree (Fig. 2) similar to the one based on the 16S rRNA genes.^5^ Therefore, *As. bogorensis* is closely related to *Go. oxydans* 621H, but distant from *Gr. bethesdensis*, suggesting that pathogenicity and the common traits of *As. bogorensis* and *Gr. bethesdensis* independently evolved during the evolutionary adaptation of the two AAB.

**Table 1.** Comparison of the *As. bogorensis* NBRC 16594 genome with other complete Acetobacteraceae genomes

Species	Strain	Plasmid	Length (bp)	GC (%)	CDS^a^	tRNA	rRNA operon	Transposase^b^	Reference	Trait
*As. bogorensis*	NBRC 16594	0	3,198,265	59.8	2,758	58^c^	5	14 (4, 9)	This work	Isolated from the flower of the orchid tree
*Gr. bethesdensis*	CGDNIH1	0	2,708,355	59.1	2,437	52	3	15 (4, 12)	Greenberg et al.^20^	Chronic granulomatous disease
*Ac. pasteurianus*	IFO 3283-01	6	3,340,249	53.1	3,050	58^c^	5	268 (52, 47)	Azuma et al.^13^	Used for brewing traditional vinegar
*Go. oxydans*	621H	5	2,922,384	60.8	2,664	56^c^	4	104 (27, 24)	Prust et al.^16^	Used for vitamin C synthesis
*Ko. medellinensis*^d^	NBRC 3288	7	3,508,936	60.6	3,195	58^c^	5	204 (24, 57)	Ogino et al.^17^	Isolated from vinegar
*Ga. diazotrophicus*	PAl 5	1	3,914,947	66.3	3,501	56^c^	4	129 (22, 26)	Giongo et al. ^19^	Endophyte of sugarcane

*As.*: *Asaia*; *Gr*.: *Granulibacter*; *Ac*.: *Acetobacter*; *Go*.: *Gluconobacter*; *Ko*.: *Komagataeibacter*; *Ga*.: *Gluconacetobacter*.

^a^As per the annotation of the public sequences.

^b^The number of transposase genes. Numbers in the parentheses represent the copy number of the largest family and the number of families.

^c^One tRNA (Arg) is split by a sequence homologous to a group I intron.

^d^Formerly known as *Gluconacetobacter xylinus* NBRC 3288.^2^

### 3.2. Characteristic genes in the *As. bogorensis* genome

To clarify the characteristic genes, especially potentially pathogenic genes (described as pathogenic genes below), data from the phylogenetic profiles were used (Supplementary Table S3). Several genes are likely to be associated with pathogenicity of *As. bogorensis* in the gene families of only two pathogenic AAB (*As. bogorensis* and *Gr. bethesdensis*), and in three AAB able to habitat in eukaryotic organisms (*As. bogorensis*, *Gr. bethesdensis*, and *Ga. diazotrophicus*; Table 2). For example, *As. bogorensis* and *Gr. bethesdensis* contained genes encoding a large adhesin protein and its associated haemolysin activator protein, peroxidase genes, catalase genes, and antibiotic-resistant genes (beta-lactamases and penicillin amidase), as well as a type II secretion system. The type II secretion system and the large adhesin family genes are prevalent among a wide range of animal and plant pathogens.^46,47^ Interestingly, the *As. bogorensis* genome contained two complete operons for the *bo_3_*-type ubiquinol oxidase, which is described below (see Section 3.4). The *As. bogorensis* genome also uniquely contained an operon with two genes that were homologous to rhodopsin and beta-carotene 15,15′-monooxygenase, which may function as a light-driven ion pump and produce a retinal cofactor of rhodopsin, respectively. *Asaia bogorensis* and *Ko. medellinensis* are known to produce cellulose fibres,^17,48^ and both contained a cellulose synthase *bcs* operon (synonyms: *cel* and *ces*) composed of *bcsAB*, *bcsC*, and *bcsD* genes (Asbog_01433–Asbog_01431). Those operons were classified into the gene families conserved in only *As. bogorensis* and *Ko. medellinensis* (Supplementary Table S3A).

**Table 2.** Summary of characteristic gene content of *As. bogorensis* NBRC 16594 among six Acetobacteraceae genomes

Species	Strain	Large adhesin	Peroxidase	Catalase	Penicillin amidase	Beta-lactamase^a^	Type II secretion	*cyoABCD*
*As. bogorensis*	NBRC 16594	1	1	2	1	3	1	2
*Gr. bethesdensis*	CGDNIH1	1	1	3	1	3	1	1^b^
*Ga. diazotrophicus*	PAl 5	0	0	3	0	4	1	1
*Ac. pasteurianus*	IFO 3283-01	0	0	1	0	2	0	1^b^
*Go. oxydans*	621H	0	0	1	0	2	0	1
*Ko. medellinensis*	NBRC 3288	0	0	1	0	2	0	1

*As.*: *Asaia*; *Gr*.: *Granulibacter*; *Ga*.: *Gluconacetobacter*; *Ac*.: *Acetobacter*; *Go*.: *Gluconobacter*; *Ko*.: *Komagataeibacter*.

^a^Beta-lactamase family (PFAM accession PF13354.1).

^b^Contains another terminal oxidase, a cytochrome *c* oxidase, as previously reported.^45^

### 3.3. Transcriptome analysis

**Figure 3.**
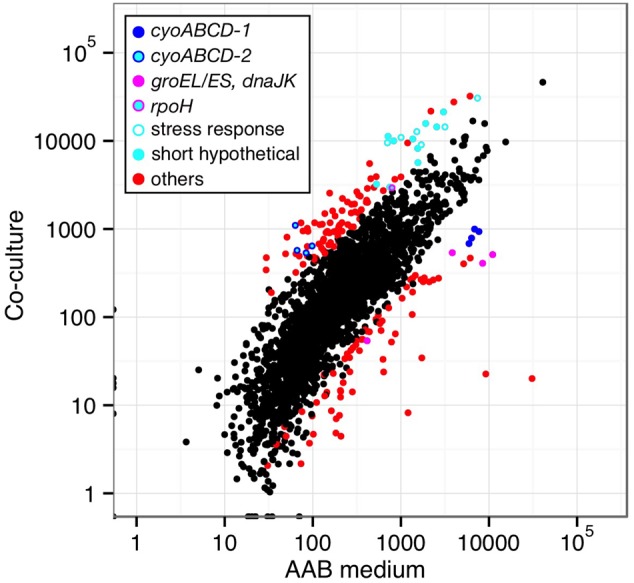
Comparison of whole RNA-seq results of different culture conditions. RPKM values from RNA-seq analyses under the ‘AAB’ and ‘co-culture conditions’ were plotted as *X*–*Y* axes for each gene. Coloured dots indicate genes showing significantly different expression (*P* = 0.001, listed in Supplementary Table S4), and categories are shown inside of the graph.

*Asaia bogorensis* and *Am. methanolica* have been reported as opportunistic human pathogens.^8–10,12^ *Asaia bogorensis* NBRC 16594 and *Am. methanolica* NBRC104435 were shown to grow at 37°C under ‘tissue culture conditions’ (Supplementary Fig. S1).^23^ To identify *As. bogorensis* genes that contribute to its growth in human bodies, comparative transcriptome analysis by RNA-seq was performed using *As. bogorensis* cultured under three different conditions: ‘co-culture conditions’, involving tissue culture at 37°C with mammalian cells, ‘tissue culture conditions’, involving the same culture conditions at 37°C but without mammalian cells, and in a YPGD medium at 30°C (‘AAB condition’). The results for ‘co-culture conditions’ and ‘tissue culture conditions’ showed high reproducibility for RNA-seq experiments (Supplementary Fig. S2). For the 2,833 genes of *As. bogorensis*, the expression of 101 and 78 genes was significantly higher and lower under ‘co-culture conditions’ than under the ‘AAB condition’, respectively (Fig. 3 and Supplementary Table S4). Individual results for genes that encode terminal oxidases, primary dehydrogenases, genes that are unique to *As. bogorensis*, and genes related to stress responses are described below.

### 3.4. Genes for electron transport chain

**Figure 4.**
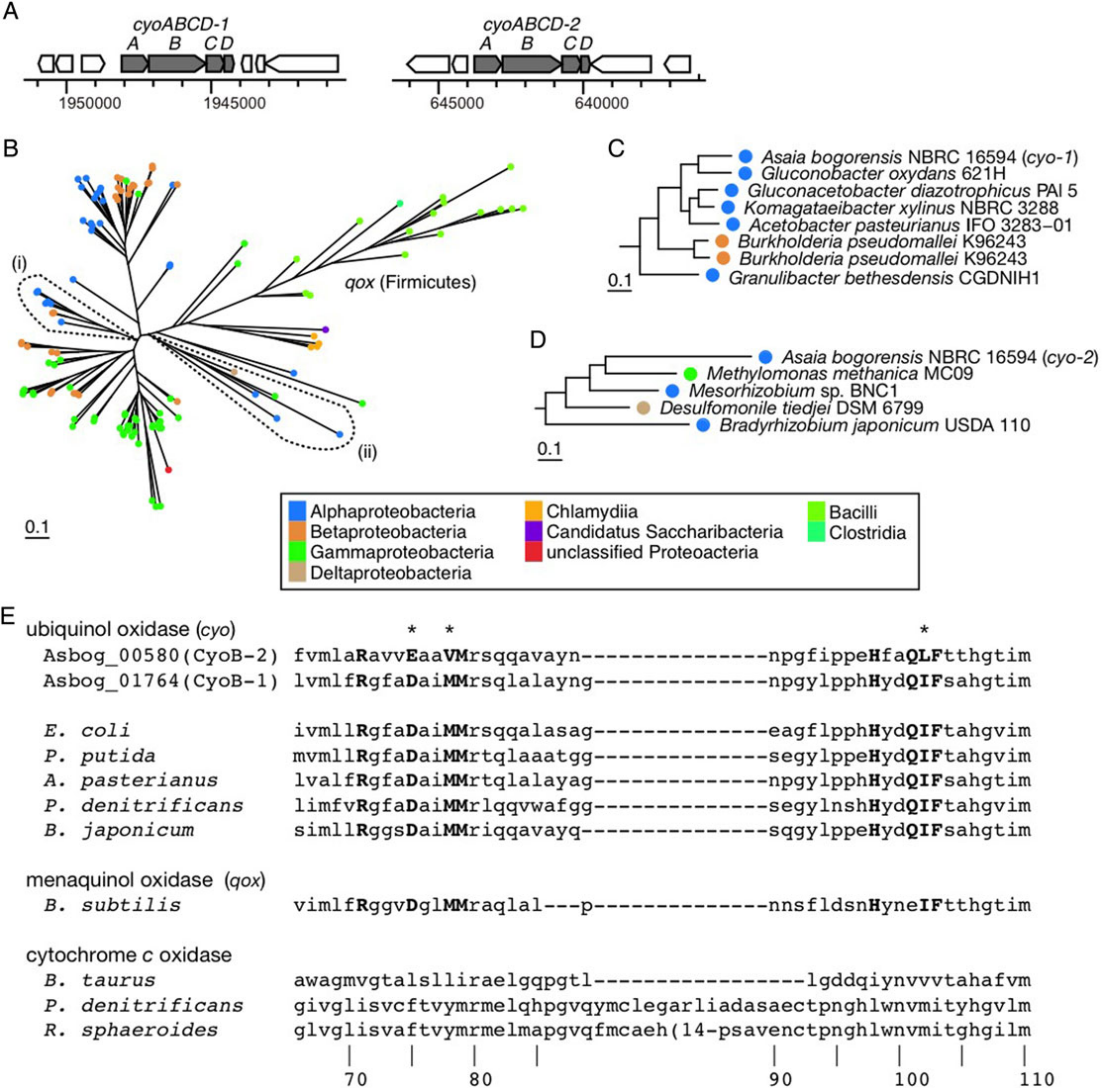
Cytochrome *bo_3_*-type ubiquinol oxidases. (A) Genetic organization of *cyoABCD-1* and *cyoABCD-2* operons of *As. bogorensis.* (B) The phylogenetic analysis based on an alignment of a concatenated amino acid sequence of four genes, *cyoABCD*. Clades (i) and (ii) as indicated by broken lines include *cyoABCD-1* and *cyoABCD-2*, respectively. (C and D) enlarged views of the clades (i) and (ii), respectively. (E) Putative ubiquinol-binding site. Residues in bold denote the conserved residues for the ubiquinol-binding site.^49^ Asterisks denote alteration of residues in *As. bogorensis cyoB-2*. The numbers indicate amino acid positions of *Pseudomonas denitrificans* cytochrome *aa_3_* (PDB 1AR1).

Two complete operons of *cyoABCD* encoding *bo_3_*-type ubiquinol oxidases were identified in the *As. bogorensis* genome and assigned as *cyoABCD-1* (Asbog_01765–Asbog_01762) and *cyoABCD-2* (Asbog_00581–Asbog_00578) (Table 2 and Fig. 4A). Within 705 prokaryotic species that represent 705 different genera (Supplementary Tables S1 and S2), 98 species contained *cyoABCD* genes, but only eight, besides *As. bogorensis*, contained two or more complete *cyo* operons. Most of the eight species are animal and plant pathogens or symbionts, including *Bordetella bronchiseptica* RB50, *Burkholderia pseudomallei* K96243, *Stenotrophomonas maltophilia* K279a, and *Ralstonia solanacearum* GMI100. Other AAB contained only one operon for the oxidase in each genome. The *cyoABCD-1* operon belonged to a monophyletic clade formed by *cyoABCD* genes from the other AAB (Fig. 4B and C), whereas the *cyoABCD-2* operon belonged to a clade composed of various *Proteobacteria* (Fig. 4B and D). Genes encoding haeme O synthase, *cyoE*, and assembly factor *surf1* were not in the flanking regions for both operons.

**Figure 5.**
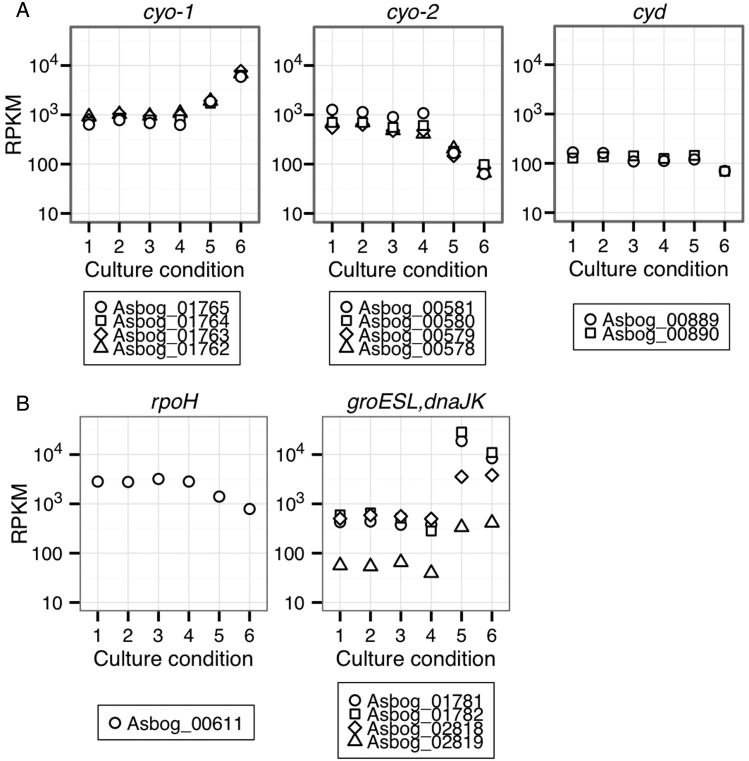
Gene expression in different conditions. (A) Ten genes that encode subunits of terminal oxidases. (B) Five heat shock genes. In every panel, lanes 1–5 indicate various conditions (lanes 1 and 2, co-culture with HEK293; lanes 3 and 4, co-culture with McCoy; lane 5, ‘tissue culture condition’; lane 6, ‘AAB condition’), and vertical axis shows values of RPKM.

The structural and mutagenic studies performed on the *bo_3_*-type ubiquinol oxidase of *Escherichia coli* strongly suggest that the ubiquinol-binding site is in the transmembrane helices I and II, which contain a cluster of conserved polar residues in the subunit I, CyoB.^49^ Most of the residues conserved among ubiquinol oxidase CyoB were also conserved in both CyoB-1 and CyoB-2, except for three residues, 75E, 78V, and 102L, in CyoB-2 (Fig. 4E). Therefore, both CyoB-1 and CyoB-2 may be functional if those genes are expressed properly. Comparative transcriptome analysis indicated that *As. bogorensis* expressed lower levels of *cyoABCD-1* under ‘co-culture conditions’ than under the ‘AAB condition’, whereas the opposite was true for *cyoABCD-2* (Figs 3 and 5A). The results indicate that *cyoABCD-1* and *cyoABCD-2* could contribute to growth in the tissue culture condition by altering the expressions.

*Escherichia coli* contains *cyo* and *cyd* operons that encode cytochrome *bo_3_* oxidase and cytochrome *d* oxidase, respectively. Expression of *E. coli cyd* genes is upregulated under an oxygen-limited condition, so that the organism can survive under oxygen starvation conditions.^50^ *Asaia bogorensis* also has *cydAB* of cytochrome *bd* oxidase that belongs to the subfamily of cyanide-insensitive terminal quinol oxidases. In contrast, expression levels of *As. bogorensis cyd* genes were relatively consistent under the different conditions (Fig. 5A).

*Asaia bogorensis* and three other AAB, such as *Ac. pasteurianus*, *Ko. medellinensis*, and *Gr. bethesdensis*, contain one *nuo* operon for type I NADH dehydrogenases (Complex I), whereas *Ga. diazotrophicus* contains two, and *Go. oxydans* does not contain any. The phylogenetic analysis based on concatenated amino acid sequences encoded by six-well-conserved *nuo* genes (*nuoHIJKLN*) indicated that *nuo* operon of *As. bogorensis* and one operon of *Ga. diazotrophicus* formed a clade that was distinctive from one composed by *nuo* operons of *Ac. pasteurianus*, *Ko. medellinensis*, *Gr. bethesdensis*, and the other operon of *Ga. diazotrophicus* (Supplementary Fig. S3). Taken together with the phylogenetic analysis presented in Fig. 2, it appears that *As. bogorensis* and *Ga. diazotrophicus* gained an extra *nuo* gene, possibly by the horizontal gene transfer, and *As. bogorensis* and *Go. oxydans* lost one *nuo* operon that is common among AAB.

The *As. bogorensis* genome included three more operons for the electron transport: *ndh* (type II NADH-dehydrogenase [NDH-2]), *sdh* (membrane-associated FAD-dependent succinate dehydrogenase [complex II]), and *atp* (an F1F0-type ATP synthase [complex V]) operons. Expression of the *nuo*, *sdh*, and *atp* operons were relatively lower under ‘co-culture conditions’ than under the ‘AAB condition’, whereas *ndh* expression was higher under ‘co-culture conditions’ than the ‘AAB condition’ (Supplementary Table S4 and Fig. S4A). These results regarding *sdh* operon differed from a previous report^7^ that showed no activities were detected. It suggests that production of complex II is too low to detect biochemical activity, or that genetically it lost its function.

### 3.5. Membrane-associated primary dehydrogenases

AAB genomes contain several genes that encode membrane-associated quinone reductases coupling substrate oxidation with quinone reduction (primary dehydrogenases).^16,23^ *Asaia bogorensis* genome included genes for six membrane-associated primary dehydrogenases assigned based on subunit structures and amino acid sequences, i.e. SLDH1, GADH, GDH, GLDH, IDH (PQQ1), and LDH (Table 3).^14,22^ However, genes that encode membrane-associated ADH and aldehyde dehydrogenase (ALDH) were not found in the *As. bogorensis* genome. This result was consistent with reports that *As. bogorensis* does not oxidize ethanol to acetic acid.^5,7^

**Table 3.** Primary dehydrogenases in *As. bogorensis* NBRC 16594

Family^a^	Cofactor	Subfamily	Genes
Sorbitol dehydrogenase	FAD	SLDH	Asbog_00352–Asbog_00354
Gluconate 2-dehydrogenase	FAD	GADH	Asbog_02524–Asbog_02526
Glucose dehydrogenase	PQQ	GDH	Asbog_00903
Glycerol dehydrogenase	PQQ	GLDH	Asbog_02636 and Asbog_02637
PQQ-dependent family	PQQ	IDH^b^	Asbog_00451
d-lactate dehydrogenase	FAD	LDH	Asbog_00865
Pyruvate oxidase	TPP and FAD	POX	Asbog_02184–Asbog_02187

^a^Quinone reductases involved in oxidative fermentation are presented. Other quinone reductases that function in different processes are omitted from this table, such as FAD-dependent succinate dehydrogenase (Asbog_00368–Asbog_00371), FAD-dependent malate : quinone oxidoreductase (Asbog_01178), and proline dehydrogenase (Asbog_02561 and Asbog_00494).

^b^Former PQQ1 was biochemically determined to be inositol dehydrogenase.^51^

The pyruvate oxidase operon (Asbog_02184–Asbog_02187) was unique to *As. bogorensis* and showed features conserved in membrane-associated primary dehydrogenases. Asbog_02184 encoded thiamin- and FAD-binding dehydrogenase similar to a membrane-associated cytoplasmic protein, PoxB (Supplementary Fig. S5A and B).^52^ Asbog_02185 encoded a protein with seven transmembrane helices. Asbog_02186 encoded a protein with a domain of gluconate 2-dehydrogenase subunit 3 similar to FAD-binding small subunits of primary dehydrogenases, such as Asbog_00352 for *sldh* and Asbog_02524 for *gadh*. Asbog_02187 encoded a glucose-methanol-choline family oxidoreductase similar to FAD-binding large subunits of primary dehydrogenases, such as Asbog_00353 for *sldh* and Asbog_02525 for *gadh*. The pyruvate oxidase seems a membrane-associated primary dehydrogenase locating in periplasmic space. But, the possibility has not been excluded that the pyruvate oxidase localizes on the cytoplasmic side of the cytoplasmic membrane, because it is still very difficult to predict topological locations of proteins by bioinformatics approaches.

Interestingly, the pyruvate oxidase operon was highly expressed under ‘co-culture conditions’ comparing with other two conditions, whereas gene expression levels of the other primary dehydrogenase were not altered under the three culture conditions (Supplementary Table S4 and Figs S4B and S5C). The expression profile of pyruvate oxidase operon was similar to one of *cyo-2* under the three conditions (Fig. 5A). Alterations in gene expression for the pyruvate oxidase may be related to culturing statuses, such as nutrition, oxygen dissolved in media, and temperature, as well as interaction with mammalian cells; however, the functional implications of the alterations are unknown.

The uncharacterized PQQ-containing oxidoreductase PQQ2 subfamily (e.g. GOX1969 and Asbog_01692) partially shares its structure with BamB, which is a lipoprotein component of the BAM (beta-barrel assembly machinery) complex and catalyses the essential process of assembling outer membrane proteins.^53^ Thus, proteins in the PQQ2 subfamily were omitted from a list of primary dehydrogenases in this work. Similarly, quinone reductases involved in processes other than oxidative fermentation were omitted from the data (Table 3), such as FAD-dependent succinate dehydrogenase (Asbog_00368–Asbog_00371), FAD-dependent malate : quinone oxidoreductase (Asbog_01178), and proline dehydrogenase (Asbog_02561 and Asbog_00494).

### 3.6. Other co-culture responsive genes

To determine the most responsive genes for ‘co-culture conditions’, 30 genes that resulted in the highest expression (average RPKM ≥2,500) were selected for analysis out of 101 genes that were expressed significantly higher under ‘co-culture conditions’ than the ‘AAB condition’. Fifteen out of the 30 genes encoded hypothetical proteins with unknown functions. Interestingly, nine of the 15 hypothetical genes were relatively small (<450 bases), and eight out of the nine genes were unique to *As. bogorensis* (Fig. 3 and Supplementary Table S4A). The mapping of the RNA-seq reads to genome regions with the 15 hypothetical genes resulted in independent peaks for monocistronic genes. Expression histograms of the nine short hypothetical genes are presented in Supplementary Fig. S6.

In addition to these short hypothetical genes, there were six stress response genes in the 30 most responsive genes under ‘co-culture conditions’, including *rpoH* (Asbog_00611) for heat shock sigma factor Sigma32, and five genes for heavy-metal resistance domain protein (Asbog_00970), thioredoxin peroxidase (Asbog_01975), stress response protein, CsbD (Asbog_00532), cold shock transcriptional regulator (Asbog_02006), and a starvation-inducible DNA-binding protein/stationary phase protection protein, Dps (Asbog_00671). In contrast to the *rpoH* gene expression, *groEL*/*groES* (Asbog_01781 and Asbog_01782) and *dnaJK* (Asbog_02819 and Asbog_02818) were expressed significantly less under ‘co-culture conditions’ than under ‘AAB conditions’ (Fig. 5B and Supplementary Table S4B). This discordance of gene expression between *rpoH* and its prospected regulons is unknown at this time.

## 4. Discussion

*Asaia bogorensis* is a unique member of AAB and was reported as an opportunistic pathogen that causes human peritonitis and bacteraemia. Here, we determined the complete genome sequence of an *As. bogorensis* type strain NBRC 16594, which was isolated from flowers and fruits. The comparative genome analyses with other complete genomes showed that the bacterium genome contained putative pathogenic genes, including genes encoding type II secretion system and large cell adhesin, and genes responsible for elimination of reactive oxygen species and antibiotics. The genome data may provide basic information for further characterization of *As. bogorensis* pathogenicity using clinical isolates.

RNA-seq analyses using various culture conditions illuminated that expression of many short hypothetical genes, most of which are unique to *As. bogorensis*, were significantly higher under the ‘co-culture conditions’ with two types of mammal cells than under the ‘AAB condition’. It was previously reported that small proteins were involved in the adaptation to stressful environments,^54,55^ and thus those short hypothetical genes may response to stressors under the ‘co-culture conditions’. Expression levels of certain stress-responsive genes were also significantly high under the ‘co-culture conditions’, including genes encoding RpoH, CsbD, Dps, heavy-metal resistance domain proteins, thioredoxin peroxidase, and cold shock transcriptional regulator. Most of the AAB are mesophilic and can grow only at 35°C or below. But certain AAB species, including *As. bogorensis* and *Ac. pasteurianus*, could be adapted to higher temperature environments managing those stress-responsive genes. *Asaia bogorensis* increased gene expression of RpoH under the ‘co-culture conditions’. However, gene expression of the assumable regulon of RpoH, including typical heat shock protein (HSP) genes, *groEL*/*groES* and *dnaJK*, were significantly lower under ‘co-culture conditions’. Many reports showed that bacterial HSPs stimulate immune responses of their hosts and host cells produce proinflammatory cytokines leading to inflammation.^56,57^ The low expression of HSPs under the ‘co-culture conditions’ may provide a clue to understand how *As. bogorensis* avoids immunosurveillance and establishes chronic infection in human bodies.

Two *cyo* operons were identified in the *As. bogorensis* genome and showed opposite expression profiles under different conditions. There are five draft genomes of *Asaia* species in the public database, including *Asaia astilbis* JCM 15831 (GCF_000613845.1), *As.* sp. SF2.1 (NCBI assembly ID GCF_000505765.1),^58^ *Asaia prunellae* JCM 25354 (GCF_000613885.1), *As. platycodi* JCM 25414 (GCF_000614545.1), and *As. platycodi* SF2.1 (GCF_000724025.1).^22^ All *Asaia* species, except *As. astilbis*, have two *cyo* operons that correspond to *cyoABCD-1* and *cyoABCD-2. Asaia astilbis* genome contained mutated *cyoABCD-1* but no *cyoABCD-2* operon. It may be because the draft genome DNA sequence was highly erroneous or because its mutation rate was high. *Asaia* species were identified in the gut and salivary gland of *Anopheles stephensi*, an Asian malarial mosquito vector.^59,60^ It is possible that generation of the two *cyo* operons in an *Asaia* ancestor was a key evolutionary event that led to the survival in a variety of environments, including high temperature conditions and inside of multicellular organisms. Besides *As. bogorensis*, only 8 species among 705 prokaryotic species contained two or more complete *cyo* operons, and most of the species are animal and plant pathogens or symbionts. Further analyses are required to clarify the evolutionary relationship among those species and the meaning of the gene expression alteration.

In conclusion, complete genome sequencing, genome comparison with other bacteria, and transcriptome analysis of *As. bogorensi*s identified genes that may confer the ability to survive in various and changing environments.

## Accession numbers

The accession number of the genome sequence and the BioProject ID of *As. bogorensis* NBRC 16594 reported in this paper is DDBJ:AP014690.1 and PRJDB519, respectively.

## Supplementary data

Supplementary data are available at www.dnaresearch.oxfordjournals.org

## Funding

This work was financially supported by the Advanced Low Carbon Technology Research and Development Program (ALCA), and a grant-in-aid for Scientific Research from the Ministry of Education, Culture, Sports, Science and Technology of Japan (KAKEN-HI: 22510222). Funding to pay the Open Access publication charges for this article was provided by the Advanced Low Carbon Technology Research and Development Program (ALCA).

## Supplementary Material

Supplementary Data
