# Supplementary material for: Complete genome and gene expression analyses of Asaia bogorensis reveal unique responses to culture with mammalian cells as a potential opportunistic human pathogen
Source: DNA Res. 2015 Sep 10;22(5):357–66. doi: 10.1093/dnares/dsv018 (PMC4596401; doi:10.1093/dnares/dsv018)
Supplement: Supplementary Data [file supp_22_5_357__index.html]

Complete genome and gene expression analyses of Asaia bogorensis reveal unique responses to culture with mammalian cells as a potential opportunistic human pathogen — Supplementary Data 

# Complete genome and gene expression analyses of *Asaia bogorensis* reveal unique responses to culture with mammalian cells as a potential opportunistic human pathogen

## Supplementary Data

Supplementary Data

- Supplementary Data - doc file
- Supplementary Figures - ppt file
- Supplementary Table 1 - xlsx file
- Supplementary Table 2 - xlsx file
- Supplementary Table 3 - xlsx file
- Supplementary Table 4A - xlsx file
- Supplementary Table 4B - xlsx file
