# Supplementary material for: Complete genome and gene expression analyses of Asaia bogorensis reveal unique responses to culture with mammalian cells as a potential opportunistic human pathogen
Source: DNA Res. 2015 Sep 10;22(5):357–66. doi: 10.1093/dnares/dsv018 (PMC4596401; doi:10.1093/dnares/dsv018)
Supplement: Supplementary Data [file supp_dsv018_dsv018supp_data.doc]

# Supplemental Figures

# Fig. S1. Viability of *A. bogorensis* NBRC 16594 on serum agar plate at 37˚C.

Eight AAB species were incubated on serum agar plate (Dulbecco’s Modified Eagle Medium F-12 (Sigma-Aldrich) with 5% fetal calf serum (Cansera) without antibiotics, 1.5% agar) at 37˚C. Ab, *Asaia bogorensis*; Am, *Acidomonas methanolica*; Ts, *Tanticharoenia sakaeratensis* NBRC 103193; Aa, *Acetobacter aceti* NBRC 14818; Ge, *Gluconacetobacter europaeus* NBRC 3261, Go: *Gluconacetobacter oxydans* 621 H, Ap: *Acetobacter pasteurianus* NBRC 3283-01; Gx, *Gluconacetobacter xylinus* NBRC 3288.

# Fig. S2. Reproducibility and Variance among RNA-seq samples.

RPKM values from 2 experiments were plotted for each gene. (A) Comparison of experimental duplicates (left, co-culture with HEK 293 cells at 37ºC; right, co-culture with McCoy cells at 37ºC). (B) Comparison between co-culture with HEK 293 cells and co-culture with McCoy cells. (C) Comparison of ‘co-culture conditions’ and ‘tissue culture conditions’ (left, co-culture with HEK 293 cells; right, co-culture with McCoy cells).

# Fig. S3. Diversity of type I NADH dehydrogenase.

(A) Phylogenetic tree of *nuo* gene products. The phylogenetic tree was constructed based of alignment of concatenated amino-acid sequences encoded by 6 *nuo* genes, *nuoHIJKLN*, from the genus-representing 706 species. Scale bar indicates substitutions per amino acid residue. Labels (i) and (ii) indicate clades of *nuo* gene products of 6 AAB, *Asaia bogorensis,* *Ac. pasteurianus*, *Ko. medellinensis*, *Gr. bethesdensis*, *Ga. diazotrophicus* and *Go. oxydans*. (B) Genetic structures of *nuo* operons of the AAB. (i) and (ii) correspond the labeled as (i) and (ii) in panel A, respectively. *Gluconobacter oxydans* 621H does not have *nuo* genes.

# Fig. S4. Expression level of genes for respiratory chains and primary dehydrogenases.

(A) Genes, *nuo*, *ndh*, *sdh*, and *atp,* for type I NADH-dehydrogenases (complex I), type II NADH-dehydrogenase (NDH-2), membrane-associated FAD-dependent succinate dehydrogenase (complex II) and an F1F0-type ATP synthase (complex V), respectively. (B) Primary dehydrogenase. lane1-5 indicate different conditions (lane1 and 2, co-culture with HEK293; lane3 and 4, co-culture with McCoy; lane 5, tissue culture condition; lane 6, AAB condition and vertical axis shows values of RPKM (reads per kilobase per million reads). Gene names and numbers are shown in each panel.

# Fig. S5. Diversity of pyruvate oxidases.

(A) Phylogenetic tree of amino-acid sequences encoded by *poxB* genes from the genus-representing 706 species. Scale bar indicates substitutions per amino acid residue. (B) Phylogenetic tree of a clade containing *As. bogorensis* pyruvate oxidase. The branches indicated by a broken line in (A) are enlarged. (C) Gene expression histogram of pyruvate dehydrogenase. Gene expression under co-culture conditions with HEK 293 and McCoy cells are shown as blue and red lines, respectively. Lines in black and magenta indicate tissue culture and AAB medium conditions, respectively.

# Fig. S6. Gene expression histograms of short hypothetical genes.

Gene locus tags are indicated in each panel. Gene expression under co-culture conditions with HEK 293 and McCoy cells are shown as blue and red lines, respectively. Lines in black and magenta indicate tissue culture and AAB medium conditions, respectively. The figure is produced using the Artemis tool equipped with BamView.

# Supplemental References

# 1. Prust, C., Hoffmeister, M., Liesegang, H., Wiezer, A., Fricke, W.F., Ehrenreich, A., Gottschalk, G. and Deppenmeier, U. (2005) Complete genome sequence of the acetic acid bacterium *Gluconobacter oxydans*. *Nature biotechnology*, 23, 195-200.

# 2. Carver, T., Harris, S.R., Berriman, M., Parkhill, J. and McQuillan, J.A. (2012) Artemis: an integrated platform for visualization and analysis of high-throughput sequence-based experimental data. *Bioinformatics*, 28, 464-469.

# 3. Carver, T., Harris, S.R., Otto, T.D., Berriman, M., Parkhill, J. and McQuillan, J.A. (2013) BamView: visualizing and interpretation of next-generation sequencing read alignments. *Brief Bioinform*, 14, 203-212.
