# Supplementary material for: Complete genome and gene expression analyses of Asaia bogorensis reveal unique responses to culture with mammalian cells as a potential opportunistic human pathogen
Source: DNA Res. 2015 Sep 10;22(5):357–66. doi: 10.1093/dnares/dsv018 (PMC4596401; doi:10.1093/dnares/dsv018)
Supplement: Supplementary Data [file supp_dsv018_dsv018supp_figures.ppt]

## Slide 1
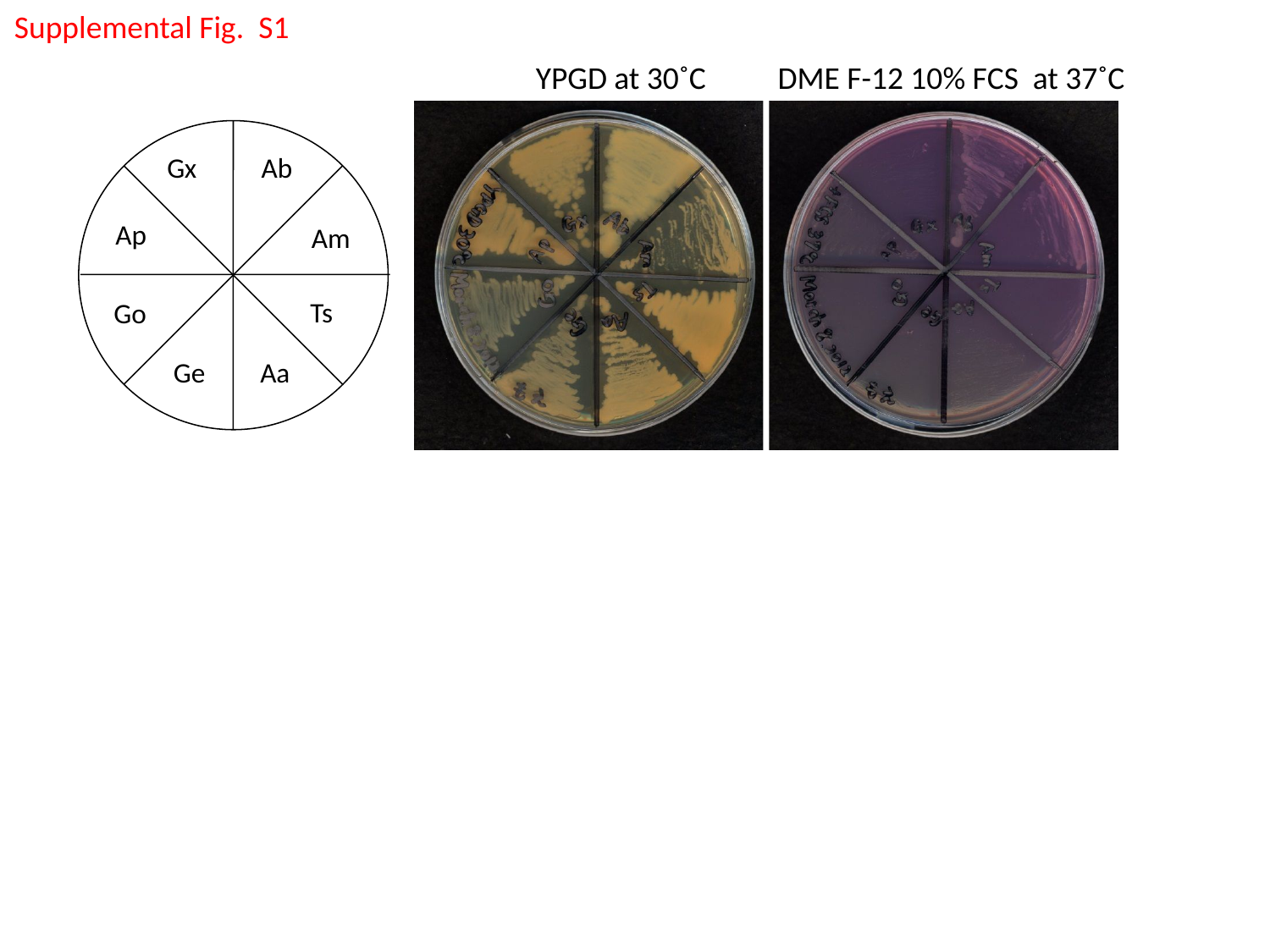

Supplemental Fig. S1
YPGD at 30˚C DME F-12 10% FCS at 37˚C
Gx
Ab
Ap
Am
Ts
Go
Ge
Aa

## Slide 2
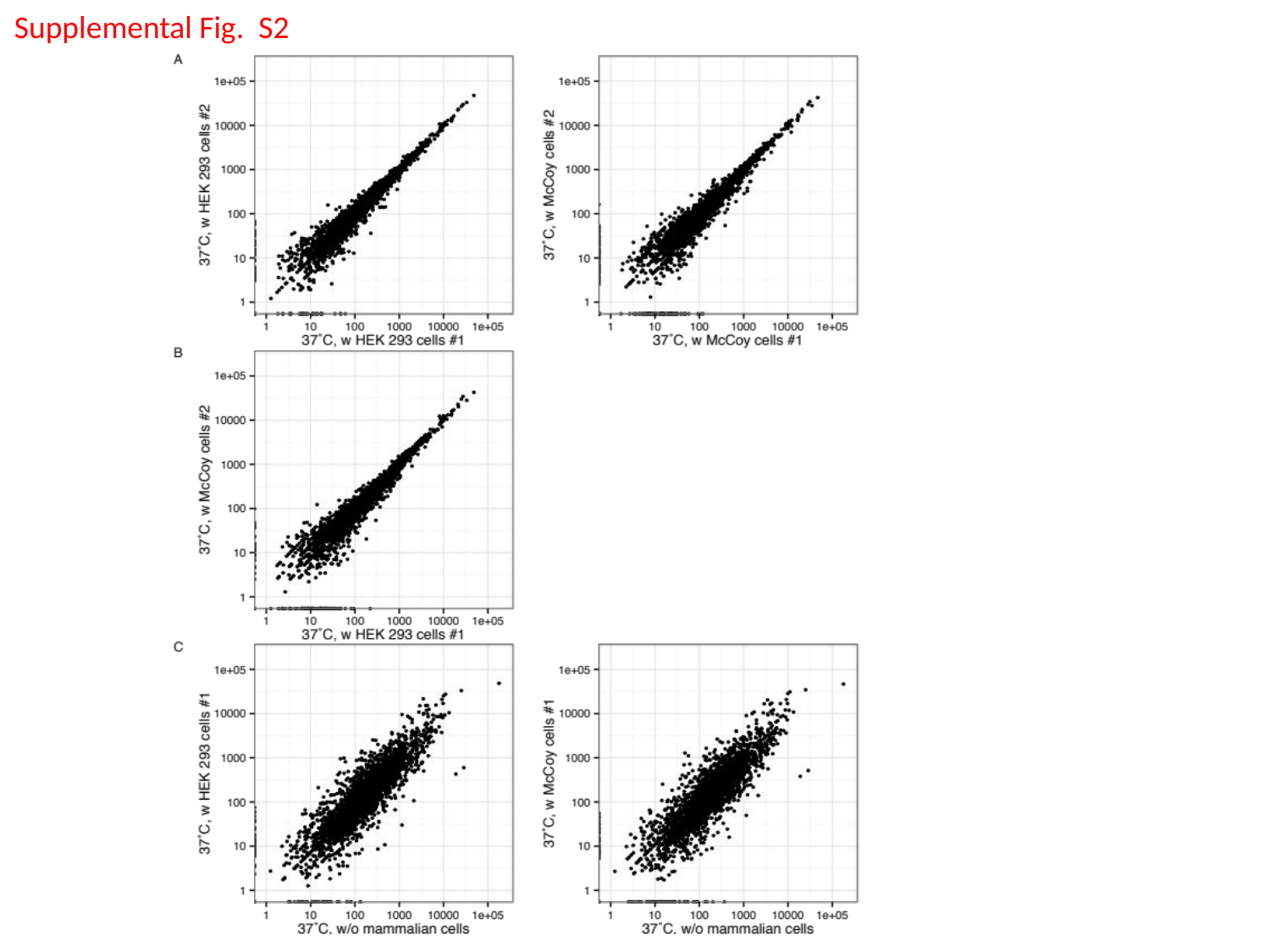

Supplemental Fig. S2

## Slide 3
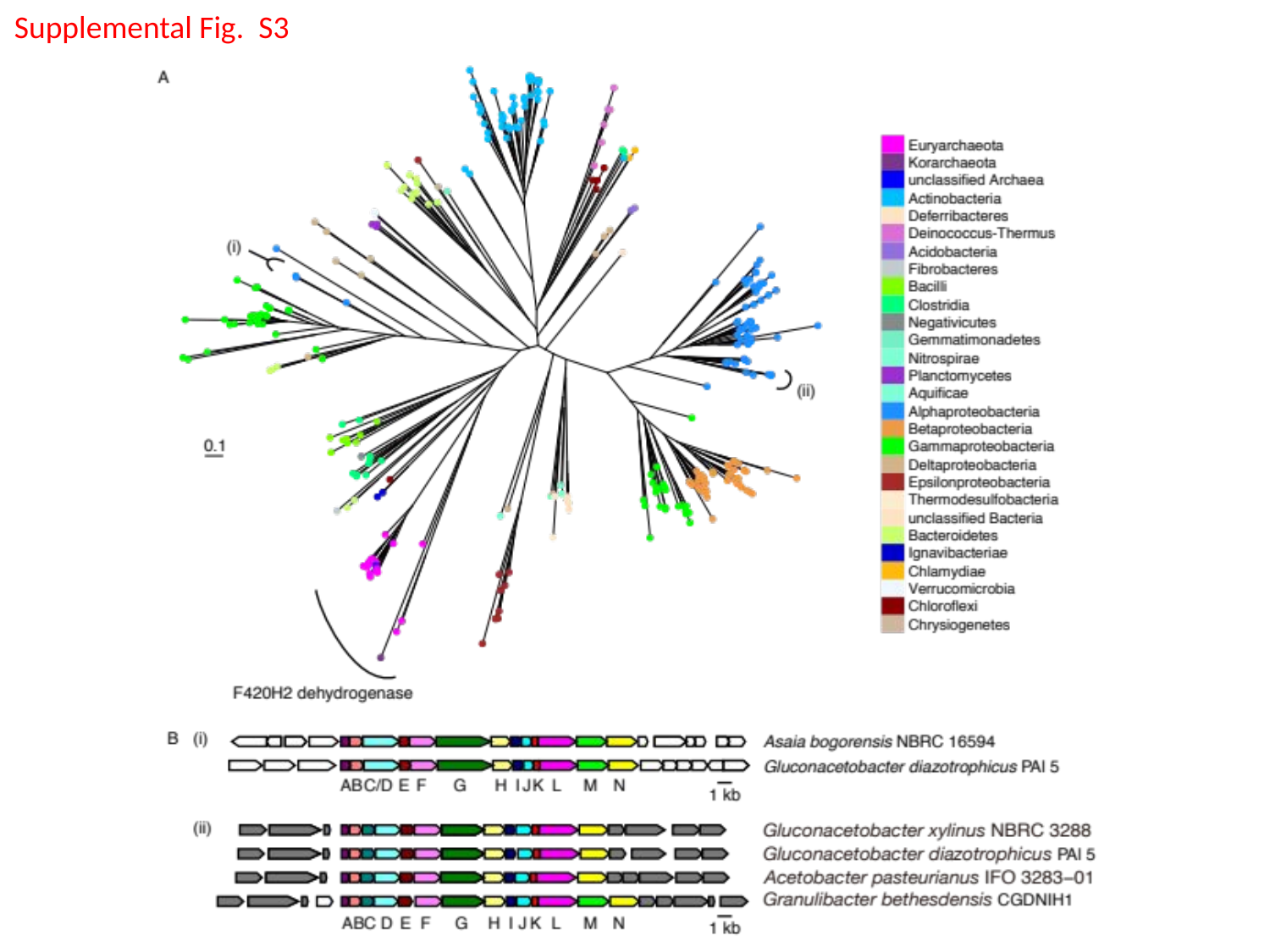

Supplemental Fig. S3

## Slide 4
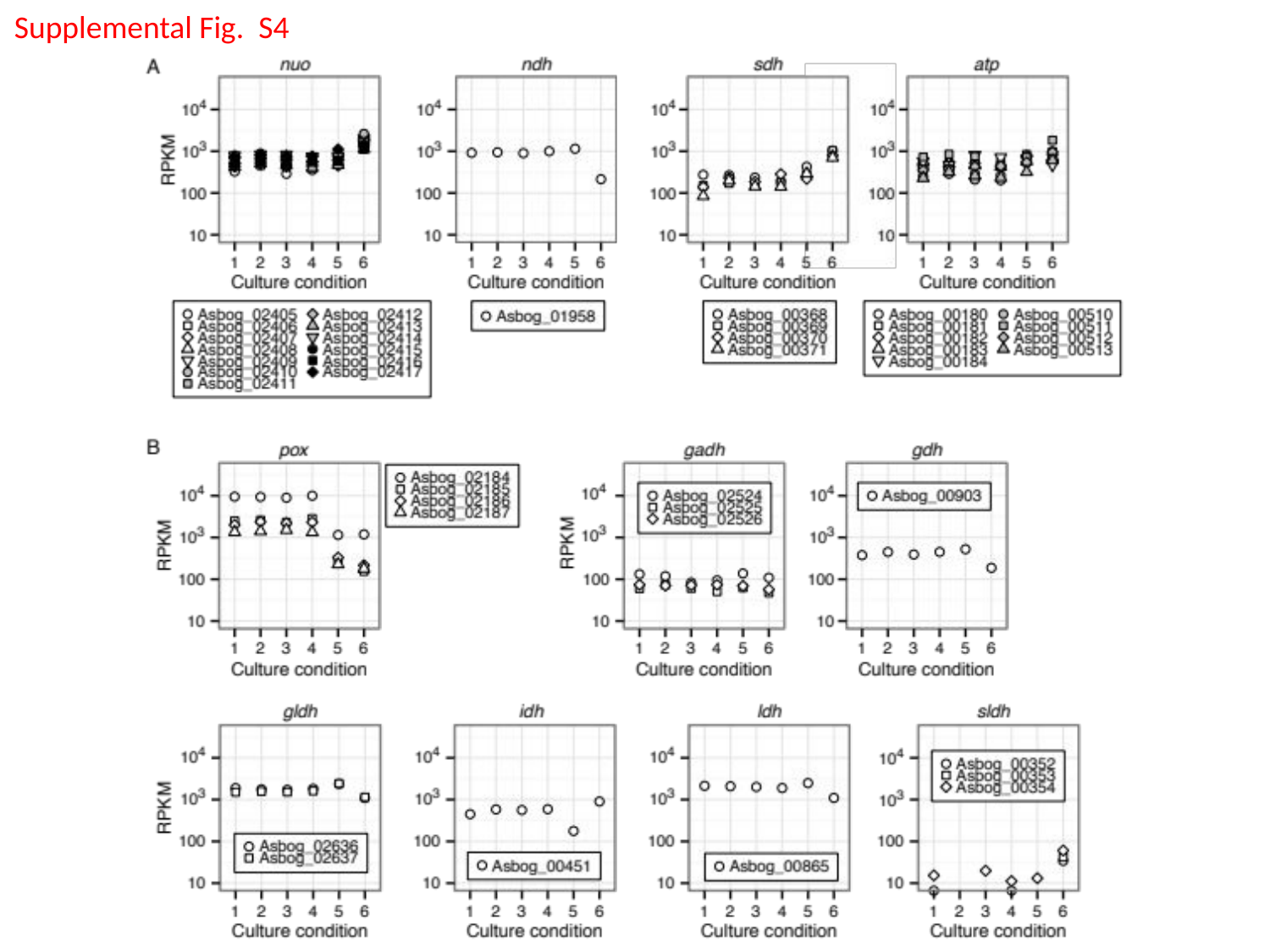

Supplemental Fig. S4

## Slide 5
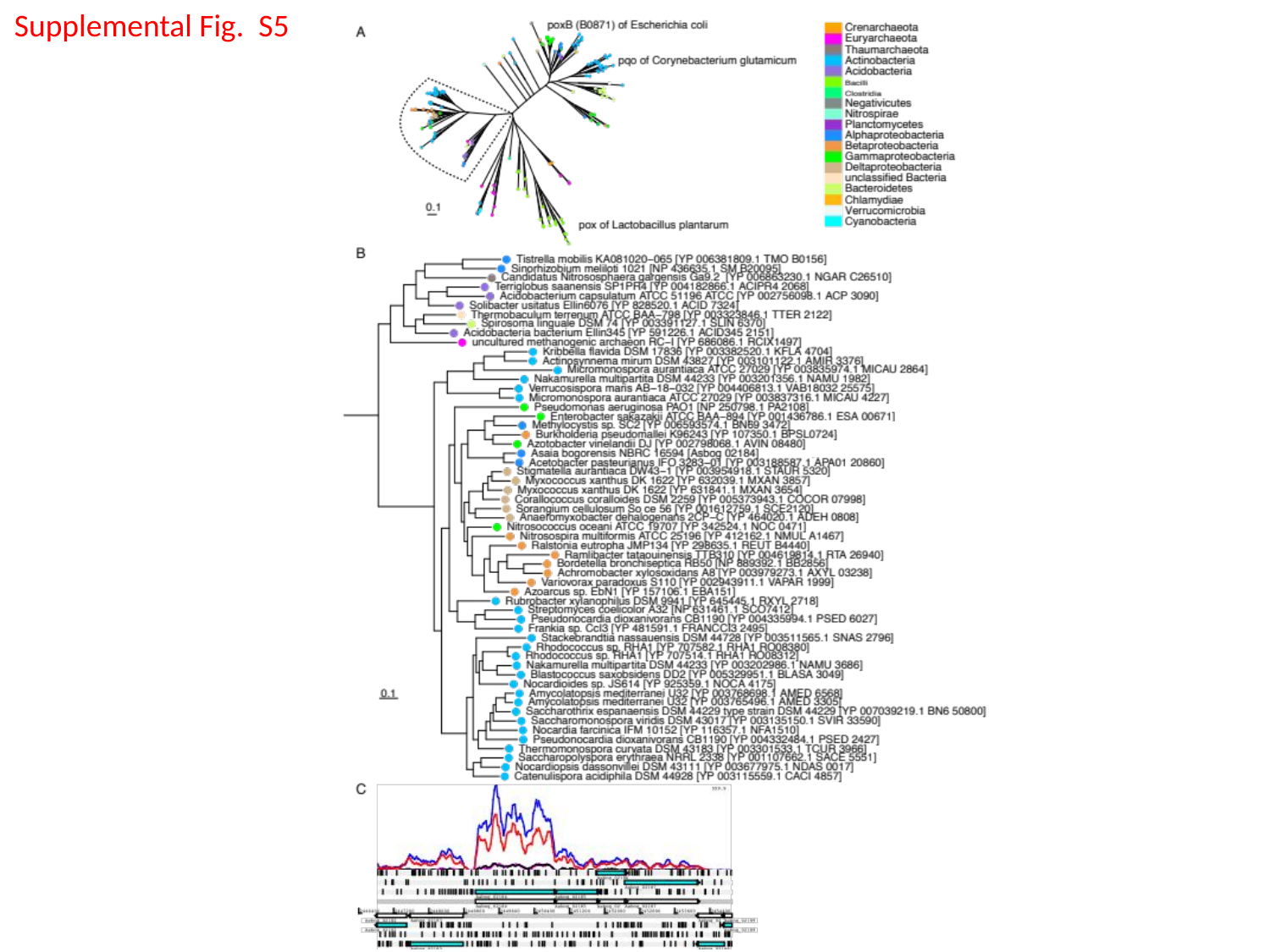

Supplemental Fig. S5

## Slide 6
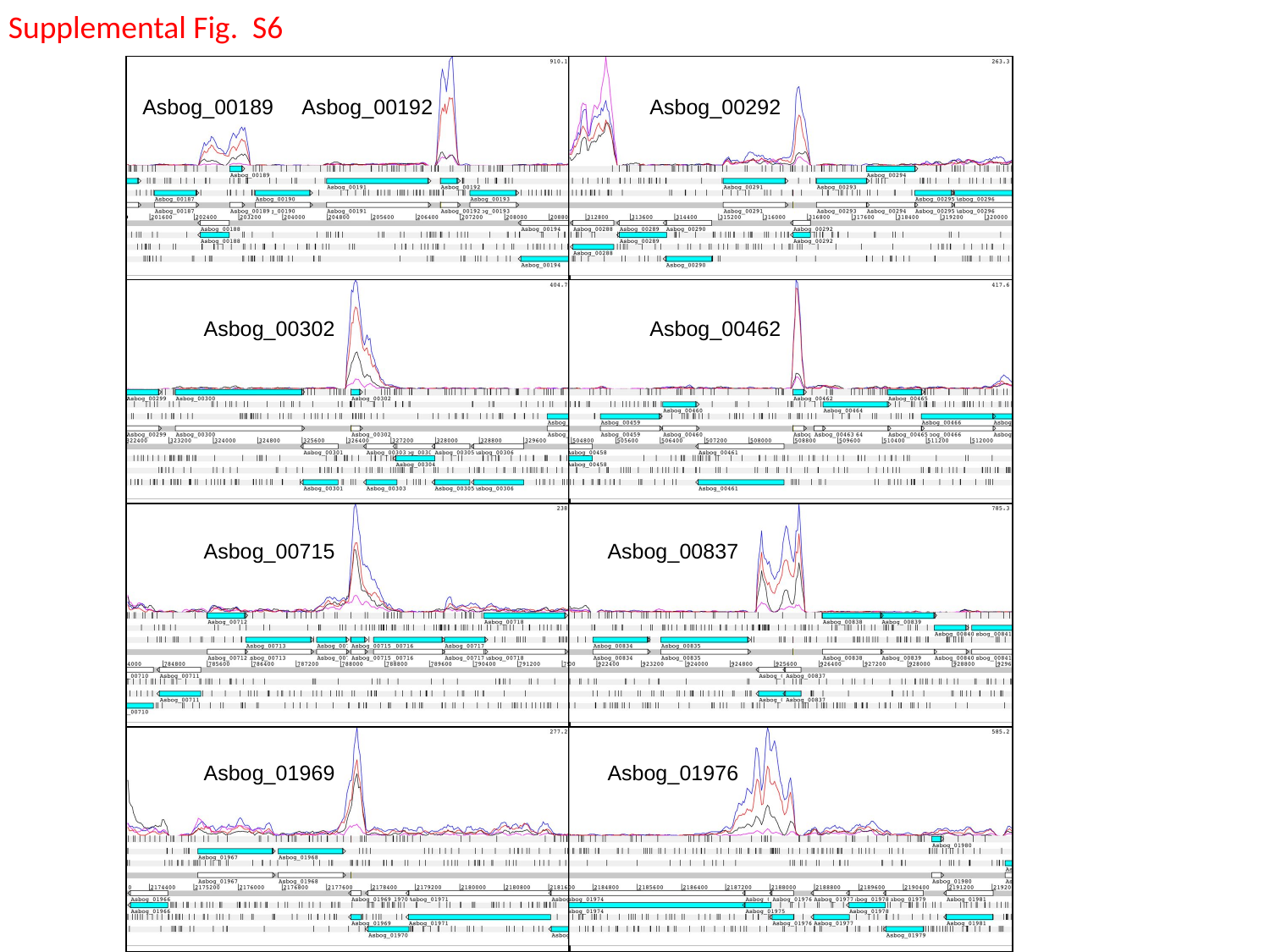

Supplemental Fig. S6
Asbog_00189
Asbog_00192
Asbog_00292
Asbog_00302
Asbog_00462
Asbog_00715
Asbog_00837
Asbog_01969
Asbog_01976
